# Application of the SF-BARI Score for Evaluating long-term Metabolic and Clinical Outcomes after Bariatric Surgery

**DOI:** 10.1007/s11695-026-08541-4

**Published:** 2026-03-26

**Authors:** Cláudia Mendes, Manuel Carvalho, João Gregório, António Palmeira

**Affiliations:** 1 Universidade de Évora, Comprehensive Health Research Centre (CHRC), Escola Superior de Enfermagem São João de Deus, Departamento de Enfermagem, Evora, Portugal; 2 Universidade Lusófona’s Research Center for Biosciences & Health Technologies Lisbon, Portugal, Lisboa, Portugal; 3 Hospital Espírito Santo de Évora - Unidade Local de Saúde do Alentejo Central, ÉVORA, Portugal; 4 CRI. COM - Centro Responsabilidade Integrada de Cirurgia da Obesidade e Metabólica, Évora, Portugal; 5 Universidade Lusófona’s Research Center for Biosciences & Health Technologies Lisbon, Portugal, Lisboa, Portugal; 6 CIDEFES, Universidade Lusófona & CIFI2D, Universidade do Porto, Lisboa, Portugal

**Keywords:** Bariatric surgery, SF-BARI score, Comorbidity remission, Roux-en-Y gastric bypass, Weight gain

## Abstract

**Background:**

Bariatric surgery is an effective treatment for severe obesity, resulting in significant weight loss and remission of obesity-related diseases. However, weight gain remains a challenge, potentially impacting long-term outcomes. The Swiss-Finnish BARIatric metabolic outcome score (SF-BARI) is a composite tool that integrates percentage total weight loss (%TWL), obesity associated disease remission, and operative complications rates to evaluate surgical optimal clinical response. This study assessed the associations between SF-BARI scores, obesity associated diseases remission, and weight gain 5 years after bariatric surgery.

**Methods:**

This retrospective cohort study included 81 patients who underwent Roux-en-Y gastric bypass (RYGB) at a Portuguese hospital, with complete 5-year follow-up data. Data on weight, body mass index (BMI), %TWL, weight gain (> 5% of nadir weight), and remission of type 2 diabetes mellitus (T2DM), hypertension, dyslipidemia, and obstructive sleep apnea (OSA) were collected at baseline and at 1 year and 5 years post-surgery. SF-BARI scores were calculated to assess outcomes. Statistical analyses included t tests, chi-square tests, and regression models to explore associations between SF-BARI scores, weight gain, and obesity associated diseases remission.

**Results:**

At 1 year, the mean BMI decreased from 44.8 ± 5.1 kg/m² to 27.7 ± 3.8 kg/m² (*p* < 0.001), with a mean SF-BARI score of 103 ± 20.3 (good). At 5 years, the BMI increased to 30.6 ± 4.7 kg/m², 57.1% of patients experienced > 5% weight gain, and the SF-BARI score decreased to 93.8 ± 25.3 (good). Obesity associated diseases remission was significant, with 60.7% of hypertensive patients, 66.7% of diabetic patients, 50% of dyslipidemic patients, and 66.7% of OSA patients having improved disease control. No significant association was found between weight gain and obesity associated diseases control (*p* > 0.05). Greater % excess weight loss was the strongest predictor of higher SF-BARI scores (r²=0.596, *p* < 0.001), whereas weight gain and higher BMI were associated with lower scores (*p* < 0.001).

**Conclusion:**

Bariatric surgery yields durable improvements in weight and obesity associated diseases control at 5 years, despite frequent weight gain. The SF-BARI score effectively captures multidimensional outcomes, and the lack of association between weight gain and obesity associated diseases remission suggests weight-independent mechanisms. However, further studies are needed to validate the prognostic value of the SF-BARI in larger cohorts.

## Introduction

Obesity has emerged as a global epidemic, affecting over 1 billion individuals worldwide and serving as a major risk factor for numerous obesity associated diseases, including type 2 diabetes mellitus (T2DM), hypertension, dyslipidemia, obstructive sleep apnea (OSA), and cardiovascular disease [1]. This chronic condition not only impairs quality of life but also imposes substantial socioeconomic burdens through increased healthcare costs and reduced productivity [2].

Metabolic and bariatric surgery is the most effective long-term treatment for severe obesity, resulting in sustained weight loss and significant improvement or remission of obesity-associated diseases. However, defining and comparing long-term surgical success remains challenging, as traditional outcome measures often rely on weight loss alone and fail to capture the full metabolic and clinical impact of surgery.

Bariatric and metabolic surgery has emerged as a potent tool in the management of metabolic diseases, particularly obesity and T2DM. Beyond its renowned efficacy in inducing weight loss, emerging evidence suggests that bariatric surgery exerts profound and enduring effects on metabolic health, often extending beyond mere weight reduction. This phenomenon has sparked considerable interest in understanding the mechanisms underlying the long-term control of metabolic diseases postsurgery and, crucially, whether these benefits persist independently of weight gain.

Procedures such as Roux-en-Y gastric bypass (RYGB) and sleeve gastrectomy (SG), represents the most effective long-term intervention for severe obesity, achieving sustained weight loss and significant remission of obesity-related diseases [3]. Large observational studies indicate durable weight loss beyond 5 years, with improvements in T2DM, lipid profiles, and other metabolic parameters, alongside reductions in overall mortality and cardiovascular events. At 5 years post-surgery, meta-analyses revealed greater weight loss and higher remission rates for T2DM and dyslipidemia following RYGB than SG.

Despite these benefits, weight gain remains a critical challenge following bariatric surgery, with prevalence rates of approximately 49% within 5–10 years and up to 72% experiencing at least 10% gain by 5 years [4, 5], particularly after RYGB (up to 64%). Weight gain is often linked to the recurrence or worsening of obesity associated diseases, undermining long-term metabolic advantages and contributing to reduced quality of life. Factors such as inadequate adherence to lifestyle modifications, anatomical changes, and hormonal adaptations contribute to this phenomenon, highlighting the need for robust tools to evaluate and predict outcomes at extended follow-up periods such as 5 years [6].

The Swiss-Finnish Bariatric Metabolic Outcome Score (SF-BARI) was specifically developed to address these limitations by integrating weight loss, remission of obesity-associated diseases, and operative complications rates into a single standardized metric. Recent evidence has demonstrated that the SF-BARI score provides a robust assessment of long-term surgical success, particularly in patients with severe obesity, including those with BMI ≥ 50 kg/m² [7]. The SF-BARI score integrates three key domains: percentage total weight loss (%TWL), improvement or remission of four major obesity associated diseases (T2DM, hypertension, dyslipidemia, and OSA), and occurrence of major operative complications, with higher values indicating superior outcomes. This score provides a comprehensive, patient-centered evaluation that correlates strongly with obesity associated diseases remission and inversely with weight gain trajectories at long-term follow-up. Studies suggest that patients who achieve higher SF-BARI scores exhibit sustained remission of obesity associated diseases and lower rates of significant weight gain (> 10% of lost weight) at 5 years post-surgery, potentially due to better metabolic adaptations and fewer reinterventions, with factors such as younger age associated with improved obesity associated diseases-related scores [8, 9]. However, the precise interplay between SF-BARI components, particularly how obesity associated diseases remission influences weight gain after 5 years, and their prognostic value in retrospective cohorts remain underexplored [10].

Importantly, emerging data suggest that improvements in metabolic disease control may persist even in the presence of partial weight regain, supporting the hypothesis that weight-independent mechanisms, such as hormonal adaptations, changes in body composition, and postoperative lifestyle interventions, contribute to long-term outcomes after bariatric surgery [11].

In this context, the present study aimed to evaluate the associations between the SF-BARI score, weight gain, and remission of obesity-associated diseases five years after RYGB. By focusing on long-term outcomes and employing a multidimensional assessment tool, this study seeks to contribute to the growing body of evidence supporting a more comprehensive evaluation of bariatric and metabolic surgery positive results, beyond weight loss alone.

## Methods

### Study Design

This article was prepared according to the Strengthening the Reporting of Observational Studies in Epidemiology (STROBE) guidelines, ensuring the transparency and completeness of our reporting of the observational study [12].

The study included adult female patients (aged ≥ 18 years) who underwent primary bariatric procedures, specifically Roux-en-Y gastric bypass (RYGB), at a Portuguese hospital.

### Data Collection

**Fig. 1:**
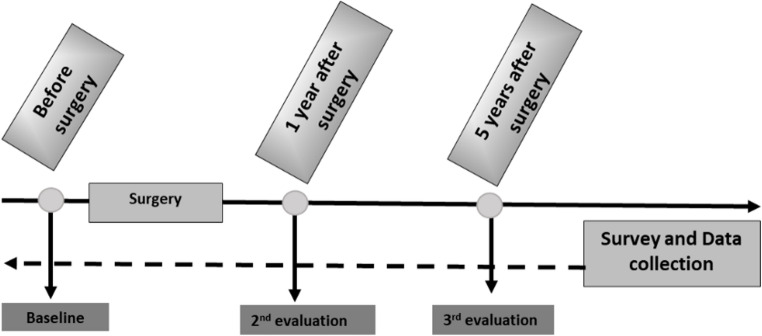
Study design and retrospective data collection points

Data collection from the electronic clinical records focused on three time points: before surgery (baseline), one year after surgery (second evaluation), and five years after surgery (third evaluation) (Fig. 1).

The data extracted included age, sex, preoperative body mass index (BMI), weight, presence of obesity-related diseases (T2DM, hypertension, dyslipidemia, and OSA), %TWL and weight gain, defined as a gain of > 5% of the lowest postoperative weight achieved (nadir weight). Obesity associated diseases status was defined according to standardized criteria: T2DM (HbA1c ≥ 6.5% or use of antidiabetic medications), hypertension (blood pressure ≥ 140/90 mmHg or antihypertensive medication use), dyslipidemia (LDL ≥ 160 mg/dL, triglycerides ≥ 200 mg/dL, or lipid-lowering therapy), and OSA (confirmed by polysomnography or continuous positive airway pressure use). Surgical details and perioperative complications were recorded.

### Eligibility Criteria

As inclusion criteria, participants had to be women with complete baseline and 5-year follow-up data, including weight measurements, obesity associated diseases status, and surgical outcomes. The exclusion criteria included revisional bariatric procedures, incomplete follow-up data at 5 years and pregnancy during follow-up.

### Sample Size

Patients were identified from a prospectively maintained institutional database and electronic medical records.

The study’s retrospective nature allowed the inclusion of all eligible patients within the study period to maximize power. The sample size was calculated by the G*power, a statistical power analysis software widely used for estimating sample size and power in biomedical research [13]. A minimum of 54 participants is necessary to enable the detection of a moderate estimated effect size of at least 0.5 standard deviations in the outcome total weight loss [14]. Two-way independent sample t-tests were performed with an alpha error of α = 0.05 and a power of 1-β = 0.95.

The hospital’s database included 384 patients with RYGB, with five years of follow-up. After applying the inclusion and non-inclusion criteria, 235 female patients were selected. Within this group, 132 patients were eligible and 84 agreed to participate in the study, whereas 78 declined or were not available to respond. The final sample included 81 females with complete answers and data.

### Outcomes

The primary outcome was the SF-BARI score at one and five years. The SF-BARI integrates the %TWL, obesity associated diseases remission rate and incidence of complications.

The secondary outcomes included the relationships between SF-BARI scores and weight gain (> 5% of nadir weight), the prevalence of weight gain, and factors influencing SF-BARI scores, such as age, sex and baseline BMI.

#### SF-BARI Score Calculation

The SF-BARI is a composite scoring system designed to standardize the evaluation of outcomes after metabolic and bariatric surgery by integrating three key domains: %TWL, remission or improvement of major obesity-related diseases, and occurrence of major operative complications. The SF-BARI score was calculated to assess comprehensive surgical outcomes. This composite score evaluates four obesity associated diseases: T2DM (HbA1c < 6.5% off medication), hypertension remission (off antihypertensive medication), dyslipidemia remission (LDL-C < 115.8 mg/dL off medication), and OSA (cessation of CPAP therapy). The SF-BARI score ranges from 100 to 200. The minimum values of the scores equal the number of deaths related to MBS complications. The total score is categorized into five outcome assessments ranging from suboptimal to excellent (Excellent ≥ 135; Very good 110 to < 135; Good 70 to < 110; Fair 35 to < 70; Suboptimal < 35).

#### Percentage Total Weight Loss (%TWL)

This domain evaluates weight loss optimal clinical response based on %TWL and is calculated as follows:

$$\%TWL=\left(\frac{Initial\;Weight-Follow-up\;Weight}{Initial\;Weight}\right)\times100$$

#### Weight Gain (%)

For this study, the weight one year after surgery was considered the minimum postoperative weight. There is considerable debate about establishing the appropriate threshold to predict weight gain [4, 5, 9, 15–17].

The definition of weight regain after bariatric surgery remains heterogeneous, with clinically relevant thresholds ranging from 5% to 15% or higher across studies. In the present study, a ≥ 5% weight gain cutoff was deliberately adopted as a conservative and sensitive criterion to capture early and moderate weight regain rather than severe metabolic relapse. This approach allowed for the identification of subtle long-term weight trajectories within five years after surgery but may underestimate the impact of more pronounced or progressive weight gain, calculated via the formula: [(Present weight – minimum postoperative weight)/(Preoperative weight − minimum postoperative weight)]*100.

#### Obesity Associated Diseases Control

This domain assesses the status of four major obesity-related diseases: T2DM, hypertension, dyslipidemia, and OSA. For the variable controlling medical diseases associated with obesity, four categories were defined: disease control without medication, disease control with medication, uncontrolled disease without medication, and uncontrolled disease with medication.

### Ethics Considerations

The hospital Institutional Ethics Committee approved the study and adhered to the Declaration of Helsinki, and informed consent was obtained from the patients who agreed to participate.

### Statistical Analysis

Given the retrospective design and limited sample size, all regression analyses were conducted for exploratory and descriptive purposes only and were not intended to support causal or confirmatory inference. Continuous variables are presented as the mean ± standard deviation (SD) or median (interquartile range, IQR) as appropriate. Categorical variables are expressed as counts and percentages. Comparisons between groups (e.g., patients with vs. without weight gain) were performed via ANOVA for repeated measures, Student’s t test or the Mann–Whitney U test for continuous variables and the χ² test or Fisher’s exact test for categorical variables. Correlations between the SF-BARI score, weight gain, and remission of obesity associated diseases were assessed via Pearson or Spearman correlation coefficients. Multivariate logistic regression was performed to identify independent predictors of high SF-BARI scores and sustained obesity associated diseases remission. A p-value < 0.05 was considered to indicate statistical significance.

## Results

A total of 81 female patients with a mean age of 49.5 ± 8.6 years were included, and all patients underwent Roux-en-Y gastric bypass more than 5 years prior to data collection. At baseline, the mean BMI was 44.8 ± 5.03 kg/m². Hypertension was present in more than one-third of the cohort, and nearly one-quarter had type 2 diabetes. Dyslipidemia and obstructive sleep apnea were also frequent obesity associated diseases.

Substantial weight reduction was achieved at 1 year post-surgery, with the mean weight decreasing from 112 ± 17.2 kg to 74.9 ± 9.9 kg (*p* < 0.001), corresponding to a mean BMI reduction from 44.8 ± 5.1 to 27.7 ± 3.8 kg/m². At 5 years, the mean weight increased slightly to 77.7 ± 12.9 kg, and the BMI increased to 30.8 ± 4.8 kg/m² (both *p* < 0.001 vs. baseline). Despite this partial gain, 49.4% of patients maintained a BMI < 30 kg/m² at 5 years. Overall, 57.1% of patients experienced > 5% weight gain between year 1 and year 5 (Table 1).

Glycemia significantly improved at 1 year (85.4 ± 8.7 mg/dL vs. 94.0 ± 23.0 mg/dL at baseline, *p* < 0.001) but partially increased by 5 years (92.2 ± 18.5 mg/dL, although it was still lower than that at baseline, *p* < 0.001). The mean blood pressure decreased from 96.0 ± 13.2 mmHg at baseline to 86.5 ± 8.8 mmHg at 1 year and remained stable at 5 years (86.4 ± 10.4 mmHg, *p* < 0.001). Total cholesterol initially decreased but then returned to near-baseline values at 5 years (169.2 ± 40.9 mg/dL vs. 166.9 ± 37.3 mg/dL, *p* = 0.127) (Table 1).

**Table 1:** Evolution of metabolic markers and disease incidence over the 5-year period

Variables mean ± SD	Baseline	1-year after surgery	5-years after surgery	*p* value
Weight (kg)	112.0 (± 17.2)	74.9 (± 9.9)	77.7 (± 12.9)	< 0.001
BMI (kg/m^2^)	44.8 (± 5.1)	27.7 (± 3.8)	30.8 (± 4.8)	< 0.001
Cholesterol (mg/dL)	169.7 (± 40.9)	158.9 (± 34.6)	166.4 (± 37.8)	0.127
Glycemia (mg/dL)	94.1 (± 23.1)	85.4 (± 8.7)	92.2 (± 18.7)	< 0.001
MBP (mm Hg)	95.9 (± 13.3)	87.8 (± 8.9)	86.4 (± 10.4)	< 0.001

*BMI* Body mass index, *MBP *Mean blood pressure

**Fig. 2:**
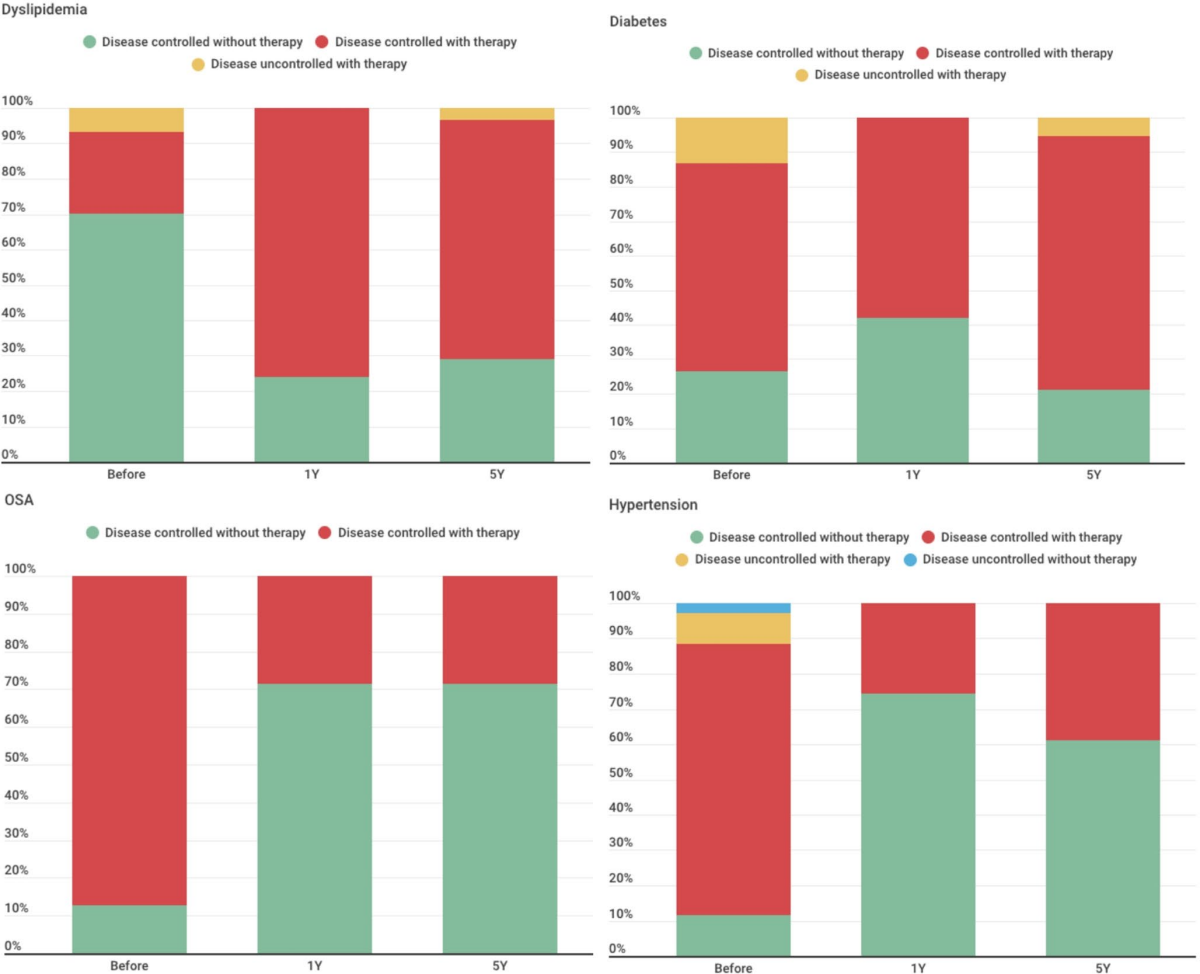
Disease control during the five years of follow-up

The prevalence of these diseases has evolved over the 5 years, with an evolution in the type of disease control. Considering the prevalence of diabetes and sleep apnea, there is only one person who benefits in terms of living without disease. However, there is a marked increase in the number of patients who can manage their obesity associated diseases without requiring additional medical or pharmacological therapy. In total, 60.7% of hypertensive patient women (*n* = 17), 66.7% of diabetic patient women (*n* = 12) and apnea patients (*n* = 4), and 50% of dyslipidemia patients (*n* = 13) saw their condition improve (defined as moving to a better disease control category). Although the absolute prevalence of certain disease-control categories declined over time, this reflects redistribution across categories rather than disease worsening at the patient level. Among patients with obesity associated diseases (*n* = 36), only 1 patient experienced worsening of diabetes control, whereas 2 patients with obesity alone (*n* = 41) were diagnosed with post surgery hypertension and dyslipidemia. In total, 37.8% of the patients (*n* = 28) experienced improvement in at least one obesity associated disease (Fig. 2).

An analysis of the results of the BMI at one year and five years after surgery revealed that 77.9% of the patients obtained a suitable result (< 30 kg/m2) at one year, but only 49.4% maintained this result at five years. With respect to weight gain, 57.1% of the patients experienced more than 5% weight gain five years after surgery.

To test whether the incidence of pharmacological control of hypertension is associated with weight gain, the chi-square test was used. There were more patients without disease and without weight gain (51.9%) than in those with weight gain (48.1%). For patients who maintained the disease but were controlled without measures, the results were different: 35.3% had no weight gain, and 64.7% had weight gain. Statistical analysis revealed that pharmacological control of hypertension is independent of weight gain (*p* = 0.453; X2 = 1.58; Fisher = 0.469) and physical activity (*p* = 0.474; X2 = 1.49; Fisher = 0.536).

In the control of dyslipidemia, 87.5% of patients with controlled disease had weight gain, whereas 12.5% did not have weight gain, and there were no significant differences between the variables (*p* = 0.139; X2 = 5.49; Fisher = 0.110).

Diabetes control is also independent of weight gain (*p* = 0.957; X2 = 0.089; Fisher = 1.000), such as OSA (*p* = 0.347; X2 = 2.12; Fisher = 0.494).

For hypertension, the probability of occurrence of each of the disease control classes was estimated from weight gain via the multinomial regression model. The probability of each disease control class was estimated as a function of weight gain (X2 = 7.75; *p* = 0.051), with no statistically significant differences. The adjusted model was not statistically significant (X2 = 1.60; *p* = 0.450). The probabilities of moving to a controlled disease with or without pharmacological measures for the “no disease” class are also not significant (*p* = 0.523; *p* = 0.238) with respect to weight gain.

Similar results have been found for dyslipidemia, diabetes and OSA. The probability of each of the disease control classes was estimated as a function of weight gain (Dyslipidemia: X2 = 6.42, *p* = 0.093; Diabetes: X2 = 0.09, *p* = 0.957; OSA: X2 = 2.88, *p* = 0.237), with no statistically significant differences. Estimates of disease control for the reference class “no disease” also reported similar results, without statistical significance.

**Fig. 3:**
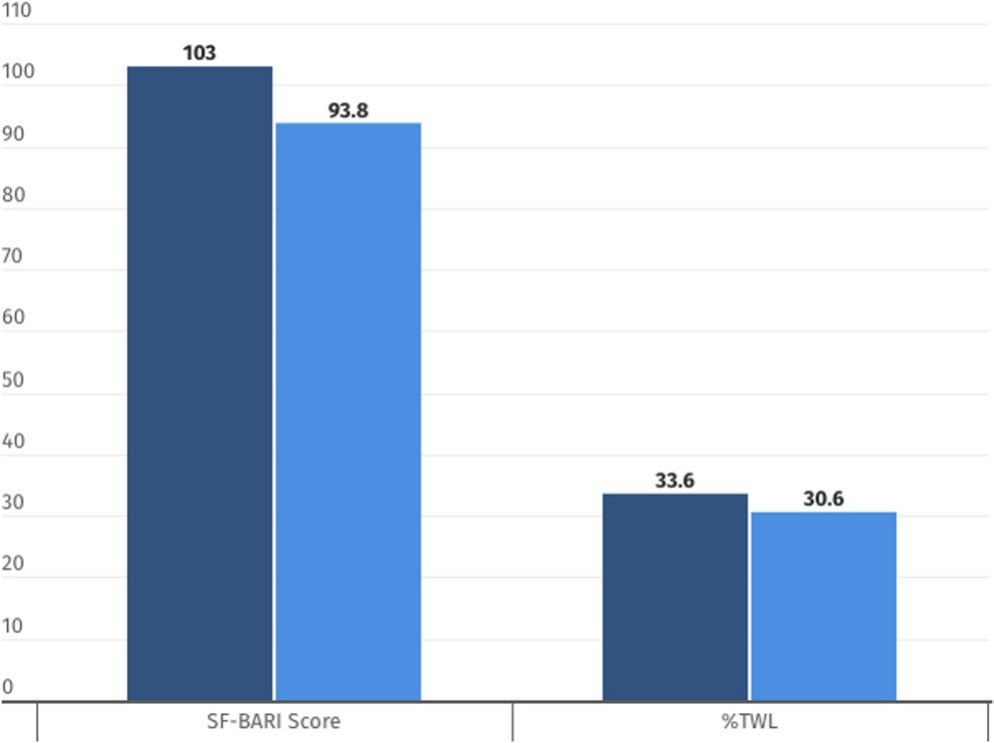
SF-Bari score and %TWL during follow-up evaluations. %TWL: Total weight loss

When stratified by SF-BARI subcategory, most patients were classified as having good or very good outcomes at both one and five years after surgery. At five years, a proportion of patients transitioned to a lower SF-BARI category, primarily reflecting partial weight regain and changes in metabolic disease control, whereas a smaller subset maintained or improved their classification. The distribution of SF-BARI subcategories at one and five years, along with corresponding weight regain and obesity-associated disease status. The mean SF-BARI score was 103 ± 20.3 at 1 year (good), decreasing slightly to 93.8 ± 25.3 at 5 years (good), which was consistent with partial weight gain and variable obesity associated diseases remission. The total weight loss (%TWL) decreased from 33.6 ± 7.9% at 1 year to 30.6 ± 9.4% at 5 years (Fig. 3).

Statistical analyses revealed no significant associations between weight gain and control of diabetes (*p* = 0.957), hypertension (*p* = 0.453), dyslipidemia (*p* = 0.139), or sleep apnea (*p* = 0.347). Multinomial regression revealed no significant predictive effect of weight gain on the likelihood of being in a specific obesity associated diseases control category across all diseases. Table 2 presents figures for the level of association between disease control and weight gain, as well as the percentages of improvement in each disease.

**Table 2:** Association between disease control and weight gain

Disease control 5 years after surgery		Weight gain			Improvement
		No	Yes	p value	%
Diabetes	Without Disease	46%	54%	< 0.957	18.5%
	Disease controlled without medication	50%	50%		
	Disease controlled with medication	50%	50%		
Hypertension	Without Disease	51.9%	48.1%	< 0.454	23.5%
	Disease controlled without medication	35.3%	64.7%		
	Disease controlled with medication	41.7%	58.3%		
Dyslipidemia	Without Disease	51.9%	48.1%	< 0.139	18.5%
	Disease controlled without medication	45%	55%		
	Disease controlled with medication	12.5%	87,5%		
	Uncontrolled disease	100%	0%		
OSA	Without Disease	43.3%	52.7%	< 0.347	7.4%
	Disease controlled without medication	60%	40%		
	Disease controlled with medication	0%	100%		

Linear regression analysis at 5 years identified % excess weight loss (PEP) as the strongest positive predictor of the SF-BARI score (r² = 0.596, *p* < 0.001, CI 0.767–1.107). Conversely, weight gain (r² = 0.309, *p* < 0.001, CI − 0.898 to − 6.06) and BMI (r² = 0.412, *p* < 0.001, CI − 4.37 to − 2.55) were both independently associated with lower SF-BARI scores. Age showed a borderline, nonsignificant negative association (r² = 0.039, *p* = 0.072). These findings suggest that long-term surgical optimal clinical response, as measured by the SF-BARI, is primarily determined by sustained weight loss and the avoidance of significant weight gain (Table 3).

**Table 3:** Linear regression analysis of 5 years after surgery and the SF-BARI score

	SF-BARI score		
	*r* ^2^	*p* value	CI 95%
Years (n)	0.039	0.072	− 1.190; 0.054
TWL (%)	0.596	< 0.001	0.767; 1.107
Weight gain (kg)	0.309	< 0.001	−0.898; −6.06
BMI (kg/m^2^)	0.412	< 0.001	−4.37; −2.55

*BMI *Body mass index, *TWL *Excess weight loss

## Discussion

This retrospective study evaluated the long-term outcomes of bariatric surgery using the SF-BARI score, with a specific focus on obesity associated diseases remission and weight gain after 5 years. Our findings demonstrated that while patients achieved significant weight loss and metabolic improvements, weight gain was common, and its association with obesity associated disease control was less straightforward.

Consistent with previous reports, we observed a substantial reduction in BMI one year after surgery, followed by partial weight gain at 5 years [16, 18]. More than half of our cohort (57.1%) experienced ≥ 5% weight gain, a rate comparable to that of long-term studies reporting that 49–64% of patients gained weight within 5–10 years [19, 20]. Despite this, many participants maintained improved control of type 2 diabetes, hypertension, dyslipidemia, and obstructive sleep apnea, which aligns with evidence that metabolic benefits may persist even in the presence of moderate weight gain [21].

Importantly, our study highlights the utility of the SF-BARI score in capturing these multidimensional outcomes. The mean SF-BARI score decreased slightly between the first and fifth postoperative years (103 vs. 93.8), reflecting both weight gain and variability in obesity associated disease remission. Nevertheless, a large proportion of patients continue to achieve “good” or better SF-BARI outcomes, underscoring the durability of bariatric surgery benefits beyond weight loss alone [7, 10].

Notably, we found no significant associations between weight gain and obesity associated disease control for diabetes, hypertension, dyslipidemia, or sleep apnea. These findings suggest that metabolic improvements may, at least partially, be mediated by mechanisms beyond weight loss, such as hormonal and gut microbiota changes [1, 22, 23]. However, our findings also revealed that patients with dyslipidemia or hypertension who required ongoing pharmacological therapy were more likely to experience weight gain, which may indicate that greater weight recovery attenuates metabolic benefits in some individuals.

Recent evidence suggests that the prevalence of metabolic related diseases such as T2DM, hypertension, and dyslipidemia among candidates for bariatric surgery is comparable to that observed in the general population. According to the World Health Organization [24], global prevalence estimates for diabetes and hypertension are approximately 10% and 22%, respectively, with dyslipidemia affecting nearly 40% of adults worldwide. Similarly to our results, studies of bariatric populations report diabetes prevalence rates of 15–25%, hypertension around 30–40%, and dyslipidemia in 30–50% of patients [3, 5, 22]. The fact that these rates are not drastically higher than global averages underscores that bariatric patients reflect the broader metabolic disease burden associated with obesity. Importantly, numerous studies have demonstrated that bariatric surgery leads to substantial remission or improvement of these conditions, particularly T2DM, through mechanisms involving weight loss, hormonal changes, and improved insulin sensitivity [25, 26]. Beyond weight loss, several mechanisms may explain the sustained remission of metabolic diseases after bariatric surgery, including improvements in insulin sensitivity, gut hormone secretion, and preservation of lean body mass. Skeletal muscle loss after procedures such as RYGB has been increasingly recognized as a potential limitation for long-term metabolic health. In this context, structured postoperative exercise has emerged as a key strategy to mitigate muscle loss and optimize functional and metabolic outcomes, as reflected by improvements in SF-BARI scores [11].

Our study has several clinical implications. First, long-term monitoring is essential, as nearly half of patients fail to maintain a BMI < 30 kg/m² at 5 years. Second, the SF-BARI score appears to be a valuable tool for assessing comprehensive surgical outcomes, integrating weight trajectories and obesity associated disease control into a single measure. Third, the independence of some cases of obesity associated disease remission from weight gain suggests the observed resolution of diabetes, hypertension, and dyslipidemia following bariatric surgery serves as strong evidence of its metabolic and cardiovascular benefits beyond weight reduction alone.

Our regression analysis further reinforces the central role of sustained weight loss in determining long-term surgical optimal clinical response. Higher % excess weight loss was the strongest positive predictor of SF-BARI scores, whereas higher BMI and weight gain were significantly associated with poorer outcomes. These findings align with recent multicenter reports showing that weight trajectories are the most critical determinant of composite surgical optimal clinical response metrics [9]. Interestingly, age showed only a borderline association, suggesting that metabolic improvements and surgical outcomes are less dependent on age than on weight control and adherence to long-term follow-up.

Our findings are in line with recent studies highlighting the value of the SF-BARI score as a comprehensive tool for evaluating long-term outcomes after metabolic and bariatric surgery. In particular, the lack of a significant association between weight regain and remission of obesity-associated diseases observed in our cohort supports the concept that metabolic benefits may be partially independent of long-term weight trajectories.

This observation is consistent with recent evidence showing that the SF-BARI score captures multidimensional surgical success beyond weight loss alone, especially in patients with severe obesity, including those with BMI ≥ 50 kg/m² [27]. Furthermore, randomized data suggest that structured postoperative exercise programs can improve SF-BARI outcomes by preserving skeletal muscle mass and enhancing metabolic health, even in the context of modest weight regain [11].

An important methodological consideration is that several variables explored in relation to the SF-BARI score, including %TWL, BMI, and weight gain, are direct components or mathematical derivatives of the composite outcome. As such, analyses involving these variables are inherently circular and violate assumptions of independence between predictors and outcome. The observed associations therefore largely reflect the internal construction of the SF-BARI score rather than independent predictive effects and should be interpreted accordingly.

Several limitations should be acknowledged. First, this was a retrospective, single-center study, which limits causal inference and may be subject to selection and information bias. Second, the study population was restricted to female patients, reflecting the characteristics of the available cohort but limiting the generalizability of the findings to male populations, in whom weight trajectories and metabolic responses after bariatric surgery may differ. The restriction to female patients’ further limits generalizability, particularly given that the SF-BARI score was developed as a sex-agnostic measure. Third, the conservative definition of weight gain (≥ 5%) and the absence of stratification by severity of regain may have attenuated the detection of associations with metabolic relapse, particularly for more severe or progressive weight gain [28]. In addition, the study was not powered for formal hypothesis testing involving the composite SF-BARI score or multivariable regression modelling. Finally, the use of SF-BARI components as explanatory variables introduces circularity, limiting the ability to formally test independent associations. Finally, quality of life outcomes, an important component of the SF-BARI, were not fully explored in this cohort.

## Conclusion

In conclusion, bariatric surgery provided significant and durable improvements in weight, obesity associated disease control, and SF-BARI scores at 5 years, despite frequent weight gain. These findings reinforce the role of the SF-BARI score as a clinically meaningful, multidimensional outcome measure and support the growing evidence that long-term metabolic benefits after bariatric surgery may occur through weight-independent mechanisms. The SF-BARI score is a practical tool for long-term evaluation, and future studies should validate its prognostic value in larger, multicenter cohorts.

## Data Availability

No datasets were generated or analysed during the current study.
